# The Relationship between Psychological Stress and Healthy Lifestyle Behaviors during COVID-19 among Students in a US Midwest University

**DOI:** 10.3390/ijerph18094752

**Published:** 2021-04-29

**Authors:** Terence Moriarty, Kelsey Bourbeau, Fabio Fontana, Scott McNamara, Michael Pereira da Silva

**Affiliations:** 1 Department of Kinesiology, University of Northern Iowa, Cedar Falls, IA 50614, USA; kelsey.bourbeau@uni.edu (K.B.); fabio.fontana@uni.edu (F.F.); scott.mcnamara@uni.edu (S.M.); 2 Faculty of Medicine, Federal University of Rio Grande, Rio Grande 90040-060, Brazil; prof.mpsilva@outlook.com

**Keywords:** COVID-19, psychological stress, physical activity, sleep, social connectedness

## Abstract

The gripping coronavirus disease (COVID-19) has imposed dramatic changes to many areas of daily living in all sectors of society across the world. We examined the relationship between perceived stress and health behaviors among college students during the COVID-19 pandemic. An online survey with measures of psychological stress, physical activity and exercise, and sleep and social connectedness was distributed in June and July of 2020. The survey was completed by 550 college students (mean age: 21.3 ± 3.8 years, 74.2% female, 94.4% Caucasian). Being female and unemployed and having a lower annual income were significantly associated with higher levels of stress. In addition, regression analysis found that reduced exercise and sleep during the COVID-19 pandemic significantly predicted the levels of stress of participants after controlling for gender, employment status, and annual income. University officials should aim to implement health-promotion strategies directed at preventing reductions in exercise and sleep duration, especially in those at greater risk of increased perceived stress such as females and economically disadvantaged students.

## 1. Introduction

Coronavirus disease (COVID-19) has various health consequences, potentially leading to cardiovascular or respiratory failure and, in some cases, death [1,2]. Aside from the health risks, the pandemic adds an array of unique barriers to regular everyday routines. Policies including stay-at-home orders, social distancing, protective mask requirements, closure of gymnasiums and bars, and restrictions to community and sporting events were set in place to reduce the rate of COVID-19 transmission. In addition, the pandemic has also created serious economic problems such as job losses, home evictions, and small business closures [3,4,5,6]. The uncertainty of the pandemic, combined with health implications and lifestyle changes, has led to increased psychological stress in countries across the globe [7,8]. 

Psychological stress is associated with various comorbidities, including cardiovascular disease, gastrointestinal problems, and depression [9,10,11]. Despite the shared struggle associated with the pandemic, the resultant psychological stress may be augmented in different demographic groups. For example, evidence suggests that women may be more vulnerable to stress than men [7,8,12,13,14,15]. In addition, unemployed individuals and individuals of a lower socioeconomic status may display higher levels of stress [8,16,17]. Furthermore, age may play a role in the development of stress, as younger adults have reported higher levels of stress during the pandemic than older adults [7,8,12,13,16]. In fact, college students have been particularly vulnerable to stress associated with COVID-19 [15,18,19,20]. The closure of university campuses, in addition to the other pandemic-induced stressors (e.g., fear of COVID-19, recent loss of a family member, agreement with the mitigation measures adopted by local and national authorities), may heighten psychological stress in this population. As university classes moved online, students reported several challenges, including difficulty concentrating, too much screen time, concerns about grades, and inadequate communication with professors [21,22]. Thus, the COVID-19 pandemic has been a significant stressor for college students.

COVID-19 can also disrupt the adoption and maintenance of healthy lifestyle behaviors such as participating in regular physical activity, obtaining adequate sleep, and socializing with friends and family. Reduced levels of physical activity during the COVID-19 pandemic have been extensively reported [13,23,24,25]. Regular physical activity is well-documented as a cornerstone of chronic disease prevention and treatment. Physical activity can lead to an array of positive health outcomes, including weight loss, improved sleep, and a strengthened immune system [26,27,28]. Aside from the aforementioned benefits, physical activity is a widely accepted stress management tool [29,30]. In contrast, physical inactivity is associated with poor mental health outcomes [31,32]. Thus, it is not surprising that higher engagement in physical activity during the COVID-19 pandemic is associated with better mental health outcomes such as lower levels of perceived stress, depression, and anxiety [13,17,23,24,25]. 

Although sleep-related issues have been extensively documented among college students prior to the COVID-19 pandemic [33,34], the pandemic has led to a further decrease in sleep quality among this population [35]. Poor sleep quality and decreased sleep duration have a negative impact on the academic achievement, quality of life, and mental health of college students [33,34]. In addition, self-reported reduction in sleep duration during the pandemic is associated with higher levels of perceived stress [13].

Finally, social distancing requirements, implemented to reduce COVID-19 transmission, may have led to the social isolation of college students. For example, college students reported increased loneliness and fewer study partners than prior to the pandemic [20,36]. Evidence from Nitschke and colleagues suggests decreased social connectedness is associated with higher levels of perceived stress [37]. Similarly, Kim and Jung note a significant positive association between social isolation and psychological distress [38]. In this regard, social connectedness may play an important role in buffering the negative mental health consequences of this pandemic. 

There is limited research regarding the adverse impact of the COVID-19 pandemic on perceived stress in college students in the United States. Furthermore, it remains to be elucidated whether the maintenance of health behaviors during the pandemic such as physical activity, sleep, and social connectedness may alleviate perceived psychological stress. Thus, the present investigation sought to examine the relationship between perceived stress and health behaviors among college students at a public university (located in the Midwest region of the United States) during the COVID-19 pandemic. Based on previous evidence, we hypothesized that lower engagement in healthy lifestyle behaviors during the COVID-19 pandemic is associated with increases in perceived stress in college students. Furthermore, we hypothesized that disruptions to lifestyle behaviors during COVID-19 are associated with higher levels of perceived psychological stress regardless of the degree to which college students engage in these behaviors prior to the pandemic. 

## 2. Materials and Methods

### 2.1. Participants and Procedures

We used non-probability sampling to select participants from a US midwestern public university during the months of June and July, 2020. The population of students in this university had the following characteristics: 58.6% of total enrolled are females, 85.6% Caucasian, 4.3% international, 2.6% Hispanic, 2.8% Black or African American, 0.9% Asian, or other or did not report race. During this time, the university had all classes in an online setting. Participants were undergraduate students who had been enrolled in a required general education course within two years prior to data collection. We invited 3302 undergraduate students by email to participate in the study. A total of 868 college students participated in the study for a response rate of 26.29%. Inclusion criteria encompassed being at least 18 years of age and a spring 2020 graduate or current undergraduate student at the university. All questionnaires were answered online via Qualtrics (Qualtrics Corp., Drive Provo, UT, USA). We acquired institutional review board approval (approval number 21-0001) and participant consent prior to data collection.

### 2.2. Instruments

#### 2.2.1. Demographic Characteristics and Behavioral Changes during the COVID-19 Crisis

Questions related to demographic characteristics and behavioral changes associated with the COVID-19 crisis were extracted from the COVID-19-related Lifestyle Change questionnaire [39]. The National Institute of Health suggested this questionnaire early in the pandemic to assist in the performance of timely behavioral research in response to the COVID-19 crisis. The demographic characteristics collected from this questionnaire were (1) age, (2) years in college, (3) major, (4) race and ethnicity, (5) employment status, (6) gender, (7) annual household income, (8) whether a friend or family member had tested positive for COVID-19, and (9) providing home care due to COVID-19. Changes in healthy lifestyle behaviors were measured with a probing question about the amount of time spent sleeping, exercising, and connecting with family and friends during the COVID-19 crisis, followed by whether each of these three behaviors increased, decreased, or remained unchanged in relation to before the COVID-19 crisis. 

#### 2.2.2. Perceived Stress Scale 4

The Perceived Stress Scale 4 (PSS-4) is a self-report measure of perceived stress in adults in relation to situations occurring in the last month [40]. The scale consists of the following four items: (1) “In the last month, how often have you felt that you were unable to control the important things in your life?”, (2) “In the last month, how often have you felt confident about your ability to handle your personal problems?”, (3) “In the last month, how often have you felt that things were going your way?”, and (4) “In the last month, how often have you felt difficulties were piling up so high that you could not overcome them?”. Each item was rated on a five-point Likert scale ranging from 0 (never) to 4 (very often). Items two and three were reverse coded. Total scores could range from 0 to 16, with higher scores reflecting higher levels of perceived stress. Scores ≥8 indicate elevated stress levels [41,42]. PSS-4 has shown adequate validity to measure perceived stress in adults [40,41,42,43]. The internal consistency of the scale was acceptable, with Cronbach’s α ranging from 0.60 to 0.82. The Spearman–Brown split-half reliability coefficient was 0.87. The PSS-4 correlated well with other measures of stress (r_s_ = 0.58–0.70). Exploratory factor analysis supported a one-factor structure of the scale accounting for 45.6 to 65.2% of the total variance. Finally, there is a strong correlation between PSS-4 and its longer counterpart PSS-10 (r = 0.95). The internal consistency of the PSS-4 in this study was adequate (α = 0.76).

#### 2.2.3. International Physical Activity Questionnaire-Short Form

The International Physical Activity Questionnaire-Short Form (IPAQ-SF) is a self-report measure of the physical activity performed by adults in the past seven days [44]. The first six items asked questions about the amount of time and days respondents engaged in vigorous, moderate, or walking types of physical activity. The last item asked respondents to identify how much time was spent sitting on a weekday. This study used the first six items to estimate the total time spent in physical activity and the last item to estimate sitting time. Craig and colleagues tested the psychometric properties of the IPAQ-SF across several countries [44]. The IPAQ-SF showed adequate psychometric properties to measure physical activity and sitting time in adults across countries. The pooled Spearman correlation across countries was 0.76 for test–retest IPAQ-SF scores and 0.30 for the correlation between IPAQ-SF scores and accelerometer data. In addition, there was approximately 80% agreement between IPAQ-SF and accelerometry in the classification of individuals meeting sufficient physical activity recommendations. Dinger, Behrens, and Han showed significant relationships between total IPAQ-SF scores and total accelerometer count (ρ = 0.23) and between total IPAQ-SF scores and pedometer steps (ρ = 0.25) [45]. In the same study, IPAQ-SF also demonstrated high test–retest reliability for total physical activity (ICC = 0.86).

### 2.3. Statistical Analysis

Descriptive statistics (i.e., mean, SD, frequencies, and percentages) were computed to examine the participants’ demographic and behavioral characteristics. Spearman–Brown correlations were computed to examine the associations between stress levels and healthy lifestyle behaviors. We also computed a hierarchical multiple regression analysis with three sequential steps. Step 1 consisted of demographic predictors such as gender, employment status, age, annual income, friends or family tested positive for COVID-19, years in college, and race. In Step 2, we tested whether behaviors such as time spent in physical activity, sitting, sleeping, and connecting with family and friends were significant predictors of stress after controlling for the demographic variables. In Step 3, we examined whether a decrease in the time spent exercising, sleeping, or connecting with friends and family after the beginning of the COVID-19 pandemic were significant predictors of stress after controlling for the demographic and behavioral characteristics. In each step, the backward procedure was used to eliminate predictors that did not significantly add to the prediction of stress. Significance was based on a value of *p* ≤ 0.05. We used IBM SPSS statistics software 24 (IBM Corp., Armonk, NY, USA) for all statistical analyses.

## 3. Results

A total of 868 college students participated in the study. We excluded the data of 318 participants from the statistical analysis (36.6%). The exclusions occurred predominantly due to incorrect (e.g., use of ranges to recall duration in physical activity) or missing data on the IPAQ questionnaire (*n* = 227). Excluded participants were not significantly different from participants included in data analyses for any of the measured demographic variables. For example, the proportion of excluded participants was not significantly different in terms of gender (χ^2^ = 2.61, *p* = 0.45) and race (χ^2^ = 6.79, *p* = 0.23) in comparison to participants included in the data analysis. The final sample consisted of 550 participants (Table 1). The mean age of the participants was 21.3 years old (SD = 3.8). The majority of the participants were female (74.2%), Caucasian (94.4%), and did not have any friends or family members positive for COVID-19 (66.2%). The majority of participants reported elevated stress levels (60.9%).

### 3.1. Healthy Lifestyle Behavior Characteristics

Most participants reported not exercising at all or exercising for less than 30 min a day (60.7%), sleeping more than 6 h a day (80.9%), and spending time connecting with friends or family (93.1%). In terms of lifestyle changes due to the COVID-19 crisis, a similar proportion of the participants reported engaging in more exercise (28.9%), less exercise (39.1%), or the same amount of exercise during the COVID-19 crisis (32%). Most participants reported that sleep duration remained unchanged during the COVID-19 pandemic (51.3%), although about a quarter of the participants reported more sleeping (27.5%), and about a fifth of the participants reported less sleeping (21.2%). A little less than a fifth of the participants reported spending less time connecting with family and friends (18.4%). Table 2 describes the healthy lifestyle behavior characteristics of the participants.

### 3.2. Association between Healthy Behavior Characteristics and Stress

Based on Spearman–Brown correlations, more time spent exercising and sleeping was significantly associated with lower levels of perceived stress (*p* < 0.05). In addition, reporting longer sitting time and larger reductions in healthy lifestyle behaviors were significantly associated with higher levels of stress (*p* < 0.01, Table 3).

During Step 1 of the hierarchical regression analysis, the results revealed that gender, employment status, and annual income were significant predictors of stress (F_3, 525_ = 19.47, *p* < 0.001, R^2^ = 0.010, adjusted R^2^ = 0.095). Being female (β = 1.84, *p* < 0.001) and unemployed (β = 0.42, *p* = 0.011) and having a lower annual income (β = −0.18, *p* = 0.001) were significantly associated with higher levels of stress. Annual income was inversely associated with levels of stress (β = −0.19, *p* = 0.001) In Step 2, total time spent exercising and sleeping were significant predictors of stress after controlling for gender, employment status, and annual income (F_5, 523_ = 18.09, *p* < 0.0001, R^2^ = 0.147, adjusted R^2^ = 0.139). Less time spent exercising (β = 0.39, *p* = 0.004) and sleeping (β = 0.76, *p* < 0.001) were associated with higher levels of stress. In Step 3, none of the healthy behavioral variables added significantly to the model after the inclusion of the healthy lifestyle change variables in the model. However, reduced exercise and sleep during the COVID-19 pandemic significantly predicted the levels of stress of participants after controlling for gender, employment status, and annual income (F_5, 523_ = 25.45, *p* < 0.0001, R^2^ = 0.196, adjusted R^2^ = 0.188). The assumption of multicollinearity was not violated in the hierarchical regression analysis (VIFs < 1.51). This model explained 19.6% of the variance in stress (Table 4). Reporting reduced exercise (β = 0.85, *p* < 0.001) and sleep (β = 1.86, *p* < 0.0001) was significantly associated with higher levels of stress. 

## 4. Discussion

The primary aim of the present study was to examine the association between perceived stress and the maintenance or adoption of healthy behaviors such as physical activity, sleep, and social connectedness during the COVID-19 pandemic among college students in the United States. The major finding of the study was that exercise participation and sleep duration are significant predictors of perceived stress in college students after controlling for demographic characteristics (gender, employment status, annual income). In addition, our results indicated that reductions in sleep and exercise were associated with higher levels of perceived stress regardless of the degree to which college students engaged in these behaviors prior to the pandemic. 

These findings support the notion that current higher levels of stress might be mitigated through achieving an adequate amount of weekly physical activity and daily sleep in this context and are consistent with pre-pandemic studies performed [46,47,48,49]. For example, VanKim and Nelson (2013) report that college students who met vigorous physical activity recommendations were less likely to report poor mental health and perceived stress than students who did not meet recommendations. Recent investigations also suggest that higher physical activity levels and longer duration of sleep are associated with lower levels of perceived stress during the COVID-19 pandemic [13,17,25]. Furthermore, results from the present study also suggest that individuals who reported a reduction in their exercise participation or sleep duration from pre- to mid-pandemic display higher levels of perceived stress. Our findings suggest that the change (reduction) in these lifestyle factors, rather than the overall accumulation of these factors, may explain the relationship between exercise participation, sleep duration, and perceived stress during the pandemic. Due to the correlational design of the current study, it is also possible that changes to the levels of pandemic-related psychological stress are driving the engagement of college students in healthy lifestyle behaviors such as exercise and sleep. Thus, to complement mental health services offered to college students, universities could implement health-promotion strategies aimed at maintaining or increasing participation in healthy lifestyle behaviors to assist college students to manage stress during crises such as the pandemic. 

Secondary findings from the present study suggest that certain groups of college students may be predisposed to heightened levels of stress. These groups specifically include female students, unemployed students, and students that earn a lower income. These findings remain consistent with previous investigations. For example, both women in the general population [7,8,12,13] and female college students [14,15] have reported higher levels of COVID-19-induced stress compared to their male counterparts. In addition, other studies have reported that the pandemic may be a more significant stressor on individuals who are unemployed or who had a lower annual income [8,16,17]. Therefore, specific wellness initiatives designed to help college students manage stress during the COVID-19 pandemic should target female and economically disadvantaged students. 

Further results from this study suggest that college students’ responses to the pandemic regarding lifestyle behaviors were not ubiquitous. It appears that the pandemic presented an opportunity for some college students to make positive changes to their lifestyle behaviors, while others made negative changes. For example, some college students reported increased participation in exercise (28.9%), sleep (27.5%), and time connecting with family and friends (46%) after the onset of the pandemic. As shown, positive changes related to social connectedness were more substantial than exercise and sleep. This may be explained by the closure of university campuses, requiring many students to return home, possibly increasing the time spent with family. In addition, the surge in the use of digital technology may also have facilitated social interactions between college students and their friends and family [50]. Conversely, other college students reported reductions in exercise (39.1%), sleep (21.2%), and time connecting with family and friends (18.4%) after the onset of the pandemic. The substantial proportion of college students reporting reductions in exercise is concerning as those who reported lower exercise levels had higher levels of perceived stress. Prevalence of elevated stress in the present study, as indicated by the PSS-4, appears slightly higher than a recent study in a similar population during the COVID-19 pandemic. Aiyer and colleagues [42] report that 48.2% (*n* = 116) of students sampled (US high-school and college students) reported elevated stress levels (PSS-4 ≥ 8) compared to 60.9% in the present study. It is clear that this is a paramount time for advocating college students to engage in physical activity in any capacity possible. Exercise and physical activity recommendations that pose a low COVID-19 transmission risk should be provided to college students. Examples of appropriate activity recommendations for individuals interested in exercising outdoors include brisk walking to class, outdoor cycling, rollerblading, and snowshoeing [51,52]. For individuals interested in exercising indoors, high-intensity interval training may be a good option, as this type of training requires little space or equipment [53].

This study is not without limitations. First, given the time sensitivity of the COVID-19 outbreak, non-probability sampling was employed. Using a randomized stratified sampling technique based on demographic characteristics such as race and gender would have improved the generalizability of the results to a more diverse population of college students. Second, self-reported changes to healthy behaviors are subject to participant bias and the ability of participants to recall information. Third, the university hosting the study had not made a decision regarding online or face-to-face classes for the fall 2020 semester at the time of this study, and thus stress may have been elevated. Considering that our final regression model only explained 19.6% of the variance in stress, additional factors certainly influence the ability of university students to cope with COVID-19-related stress. Previous research has suggested that factors such as fear of COVID-19, poor nutritional behaviors, and an abrupt switch to online academic instruction were critical stressors during the pandemic [21,54]. Finally, this study lacked pre-pandemic baseline information about healthy behaviors. The cross-sectional correlational design of the study impedes causal inferences.

## 5. Conclusions

Results from the present study and similar studies may be used to guide university officials in determining health-promotion strategies. In addition to enhancing mental health services offered to college students, health-promotion strategies should aim to prevent reductions in exercise and sleep duration, as the reduction in these behaviors was strongly associated with an increase in perceived stress. Furthermore, wellness initiatives should be designed to help students that are at a greater risk of increased perceived stress such as females and economically disadvantaged students. Finally, researchers should continue to evaluate the ongoing impact of the COVID-19 pandemic (including the subsequent lockdown restrictions and emphasis on social distancing) on health behaviors, as it is necessary to create targeted health-promotion strategies that are specific for distinct populations, such as college students.

## Figures and Tables

**Table 1:** Demographic characteristics (*n* = 550).

	Mean	SD
**Age**	21.3	3.8
**Years of college**	2.7	1.1
	***n***	**%**
**Gender**		
Male	135	24.5
Female	408	74.2
Other	7	1.3
**Race**		
White	519	94.4
Black or African American	13	2.4
Asian	14	2.5
Other	4	.7
**Annual household income**		
Less than USD 10,000	166	30.2
USD 10,000–49,999	109	19.8
USD 50,000–74,999	62	11.3
USD 75,000–99,999	67	12.2
USD 100,000–124,999	66	12.0
USD 125,000–149,999	20	3.6
USD 150,000–174,999	25	4.5
USD 175,000–199,999	11	2.0
Greater than USD 200,000	24	4.4
**Current employment status**		
Working full time	158	28.7
Working part time	246	44.7
Unemployed	58	10.5
Laid off or looking for work due to COVID-19	74	13.5
Other	14	2.5
**COVID-19 positive (friends or Family)**		
Yes	186	33.8
No	364	66.2

**Table 2:** Psychological stress and healthy lifestyle behaviors (*n* = 550).

	Mean	SD
**PSS scores**	8.1	3.0
**Total PA-IPAQ** (h/day)	1.4	1.9
**Sitting time-IPAQ** (h/day)	7.7	6.2
**Healthy Lifestyle Behaviors**	***n***	**%**
**Exercise during COVID-19 (time/day)**		
None	94	17.1
1–30 min	240	43.6
30 min–1 h	158	28.7
>1 h	58	10.5
**Exercise more or less than before COVID-19**		
More	159	28.9
Less	215	39.1
Unchanged	176	32.0
**Sleep during COVID-19 (h/day)**		
2–4 h	5	.9
4–6 h	100	18.2
6–8 h	312	56.7
8–10 h	115	20.9
10–12 h	18	3.3
**Sleeping more or less than before COVID-19**		
More	151	27.5
Less	117	21.2
Unchanged	282	51.3
**Time connecting with friends or family during COVID-19 (time/day)**		
None	38	6.9
1–30 min	243	44.2
30 min–1 h	136	24.7
>1 h	133	24.2
**Connecting with friends or family more or less than before COVID-19**		
More	253	46.0
Less	101	18.4
Unchanged	196	35.6

PSS: perceived stress scale; PA: physical activity.

**Table 3:** Associations between stress and healthy lifestyle behaviors.

	PSS Score	
	Rho	*p*
Total PA-IPAQ	–0.070	0.108
Sitting time-IPAQ	0.097	0.026
Exercise during COVID-19	–0.162	<0.001
Sleep during COVID-19	–0.217	<0.001
Time connecting with friends or family	0.012	0.787
Decreased exercise	0.192	<0.001
Decreased sleep	0.300	<0.001
Decreased time connecting with friends or family	0.154	<0.001

**Table 4:** Hierarchical regression analysis for factors associated with the PSS score (*n* = 528).

	Step 1				Step 2				Step 3			
	Initial		Final * (R²: 0.100; Adj R²: 0.095)		Initial		Final * (R²: 0.147; Adj R²: 0.139)		Initial		Final * (R²: 0.196; Adj R²: 0.188)	
	B (SE)	*p*	B (SE)	*p*	B (SE)	*p*	B (SE)	*p*	B (SE)	*p*	B (SE)	*p*
Age (years)	−0.01 (0.03)	0.856	-	-	-	-	-	-	-	-	-	-
Gender	1.83 (0.29)	<0.001	1.84 (0.28)	<0.001	1.77 (0.28)	<0.001	1.71 (0.28)	<0.001	1.63 (0.27)	<0.001	1.67 (0.27)	<0.001
Race	0.30 (0.19)	0.108	-	-	-	-	-	-	-	-	-	-
Annual income	−0.18 (0.05)	0.001	−0.19 (0.05)	0.001	−0.13 (0.05)	0.017	−0.13 (0.05)	0.013	−0.14 (0.05)	0.007	−0.15 (0.05)	0.004
Employment status	0.42 (0.15)	0.013	0.42 (0.17)	0.011	0.59 (0.17)	<0.001	0.58 (0.17)	<0.001	0.50 (0.16)	0.002	0.47 (0.16)	0.003
COVID-19 positive	−0.41 (0.26)	0.112	-	-	-	-	-	-	-	-	-	-
Total PA-IPAQ	-	-	-	-	0.01 (0.001)	0.279	-	-	-	-	-	-
Sitting time-IPAQ	-	-	-	-	0.02 (0.02)	0.297	-	-	-	-	-	-
Exercise during COVID-19	-	-	-	-	−0.48 (0.14)	0.004	−0.39 (0.13)	0.004	−0.14 (0.15)	0.341	-	-
Sleep during COVID-19	-	-	-	-	−0.74 (0.17)	<0.001	−0.76 (0.17)	<0.001	−0.21 (0.19)	0.275	-	-
Time connecting with friends or family	-	-	-	-	0.05 (0.13)	0.724	-	-	-	-	-	-
Decreased exercise	-	-	-	-	-	-	-	-	0.61 (0.28)	0.030	0.85 (0.24)	<0.001
Decreased sleep	-	-	-	-	-	-	-	-	1.62 (0.34)	<0.001	1.86 (0.28)	<0.001
Decreased time connecting with friends or family	-	-	-	-	-	-	-	-	0.54 (0.31)	0.084	-	-

*: Final models dropped non-significant variables using the backward procedure. **B**: linear regression coefficient; **SE:** standard error. **Gender:** 1 = male; 2 = female. **Race:** 1 = White; 2 = Black or African American; 3 = other. **Income:** 1 = less than USD 10,000; 2 = USD 10,000–49,999; 3 = USD 50,000–74,999; 4 = USD 75,000–99,999; 5 = USD 100,000–124,999; 6 = USD 125,000–149,999; 7 = USD 150,000–174,999; 8 = USD 175,000–199,999; 9 = greater than USD 200,000. **Employment:** 1 = working full time; 2 = working part time; 3 = unemployed/laid off or looking for work due to COVID-19. **COVID-19 positive:** 1 = no; 2 = yes. **Exercise COVID-19:** 1 = none; 2 = 1–30 min; 3 = 30 min–1 h; 4 = >1 h. **Sleep COVID-19:** 1 = 2–4 h; 2 = 4–6 h; 3 = 6–8 h; 4 = 8–10 h; 5 = 10–12 h. **Time with friends or family:** 1 = none; 2 = 1–30 min; 3 = 30 min–1 h; 4 = >1 h. **Decreased variables:** 1 = unchanged/increased; 2 = decreased.

## Data Availability

The group data presented in this study are available on request from the corresponding author. The individual data are not publicly available due to privacy and confidentiality.
